# LncRNA *GSEC* impedes the reprogramming of glucose metabolism in papillary thyroid carcinoma by inhibiting the IGF2BP2/GLUT1 axis

**DOI:** 10.20945/2359-4292-2026-0083

**Published:** 2026-07-27

**Authors:** Long Ren, Kai Zhang, Xiaotian Yu, Yong Jiang

**Affiliations:** 1 General Surgery, The Third Affiliated Hospital of Soochow University, Changzhou, China; 2 General Surgery, The Affiliated Yixing Hospital of Jiangsu University, Yixing, China; 3 Thyroid Surgery, The Third Affiliated Hospital of Soochow University, Changzhou, China; 4 Thyroid Surgery, The First People’s Hospital of Changzhou, Changzhou, China

**Keywords:** GLUT1, IGF2BP2, glucose metabolism, lncRNA *GSEC*, PTC

## Abstract

**Objective:**

To investigate the expression pattern of lncRNA *GSEC* in papillary thyroid carcinoma (PTC) and elucidate its functional role and molecular mechanism in regulating glycolytic metabolism and malignant progression.

**Materials and methods:**

The expression levels of the *GSEC* gene in thyroid cancer were determined through bioinformatics analysis of the TCGA database, and potential downstream regulatory genes were predicted. *GSEC* mRNA levels in normal thyroid cells and thyroid cancer cells were detected by qRT-PCR. Cellular functions, including proliferation, invasion, and migration, were evaluated using CCK-8 assays, Annexin-V/PI staining, western blot, EdU staining, Transwell assays, and scratch tests. Glycolytic activity was assessed by western blot, glucose uptake assays, and measurements of extracellular lactate levels. Subcutaneous and metastatic papillary thyroid carcinoma (PTC) models were established in nude mice to investigate the effects of lncRNA *GSEC* expression on tumor growth and metastasis.

**Results:**

LncRNA *GSEC* expression was significantly downregulated in thyroid cancer. Overexpression of *GSEC* in PTC-1 cells effectively inhibited their proliferation, invasion, and migration. *GSEC* downregulated GLUT1 expression by inhibiting IGF2BP2, thereby disrupting glycolysis and suppressing tumor growth and invasion. *In vivo* assay demonstrated that *GSEC* overexpression significantly reduced tumor glycolysis, retarded tumor growth, and inhibited metastasis.

**Conclusion:**

LncRNA *GSEC* is a key factor in the progression of PTC. By modulating the IGF2BP2/GLUT1 axis, *GSEC* affects cancer cell glycolysis, presenting a new potential target for metabolic intervention strategies.

## INTRODUCTION

The incidence of thyroid cancer, the most common endocrine malignancy, is sharply increasing worldwide (^1^,^2^). Papillary thyroid carcinoma (PTC) is the most frequent subtype, accounting for nearly 80% of all thyroid cancer cases (^3^). Typically, PTC is viewed as a relatively indolent malignancy. Standard treatments include thyroidectomy, radioactive iodine ablation, and thyroid-stimulating hormone (TSH) suppression therapy. These approaches generally yield a 10-year survival rate exceeding 95% (^4^). However, clinical data reveal that 5-20% of patients may still experience recurrence or develop local or distant metastases posttreatment. This recurrence rate significantly complicates the challenge of achieving a complete cure for PTC (^5^). Thus, determining the underlying mechanisms that drive PTC progression is crucial for enhancing clinical outcomes and improving patient prognosis.

Cancer cells often rewire their metabolism to support rapid growth and spread. Even when oxygen is abundant, these cells tend to break down glucose through a process called aerobic glycolysis, which is known as the “Warburg effect” (^6^). This unusual metabolic strategy not only fuels the rapid division of cells but also helps them invade surrounding tissues and migrate to distant sites. By altering cellular signaling pathways and the tumor microenvironment, this metabolic shift ultimately drives cancer progression (^7^). In PTC cells, the upregulation of key glycolytic enzymes, including PKM2, and glucose transporters, such as GLUT1 and GLUT3, suggests robust glycolytic metabolism (^8^). This metabolic shift is likely driven by the overexpression of hypoxia-inducible factor 1α (HIF-1α), which promotes glycolytic activity and supports the manifestation of the Warburg effect in PTC (^9^). However, despite indications of unusually active glycolytic metabolism in PTC, the regulatory mechanisms at work remain unclear.

Long noncoding RNAs (lncRNAs) are RNA molecules with transcripts of approximately 200 base pairs that do not encode proteins; they can regulate gene expression at multiple levels and are closely related to the development, metastasis, and prognosis of many cancers (^10^,^11^). Hence, lncRNAs have become a popular research topic in the field of cancer in recent years. Among the vast array of lncRNAs, *GSEC* has emerged as a molecule of significant interest. *GSEC* features a distinctive G-quadruplex structure that enables it to modulate gene expression and cellular functions through interactions with proteins or other RNA molecules (^12^-^14^). *GSEC*, an lncRNA, interacts with the RNA helicase *DHX36* to influence cancer cell migration and invasion (^13^); it also upregulates PFKFB3, increasing glycolytic metabolism in sepsis and driving the activation of inflammation in neutrophils (^15^). These results highlight the potential regulatory function of *GSEC* in glycolysis. Research on the role of lncRNA *GSEC* in PTC is rather scarce, and how *GSEC* affects the development of PTC and whether it is involved in the regulation of glycolytic metabolism in PTC have not yet been determined.

Motivated by the gaps in knowledge regarding lncRNA *GSEC*, our research focused on its actions within the context of PTC and its effects on glycolytic processes. Our experiments demonstrated that *GSEC* levels are notably lower in PTC tissues. Upon overexpression of *GSEC* in PTC cells, we observed a significant decrease in the activity of the IGF2BP2/GLUT1 axis, which in turn halted glycolytic reprogramming and the malignant trajectory of cancer. In summary, our study not only highlights *GSEC* as a key player in PTC progression but also reveals its molecular links to cancer cell glycolysis, paving the way for the development of *GSEC*-targeted metabolic interventions for PTC.

## MATERIALS AND METHODS

### Sample collection

A total of 5 pairs of PTC tissue samples and adjacent noncancerous tissue samples were collected for this study. All the samples were obtained from The Affiliated Yixing Hospital of Jiangsu University between 2024.12 and 2025.5. The sample collection process strictly adhered to the guidelines of the hospital’s Ethics Committee, and informed consent was obtained from all patients. The inclusion criteria were as follows: patients pathologically diagnosed with PTC; patients aged between 18 and 80 years; patients with no history of other malignant tumors; and patients who had not undergone radiotherapy or chemotherapy prior to surgery. Adjacent noncancerous tissues were collected from sites >0.8 cm away from the tumor edge. All surgically resected PTC tissues and adjacent tissue samples were processed within 30 minutes after collection. Samples intended for western blot analysis were immediately frozen in liquid nitrogen and subsequently transferred to a -80 °C freezer for long-term storage. Samples for IHC (immunohistochemistry) were fixed in 4% paraformaldehyde (PFA) and stored at 4 °C.

### Bioinformatics

We retrieved transcriptomic data for thyroid cancer (THCA) and normal thyroid tissues from the TCGA (https://portal.gdc.cancer.gov/) and GTEx (https://www.gtexportal.org) databases, which included 425 normal and 513 cancer samples. Differential expression analysis was executed using the “edgeR” package, with |logFC| > 1.5 and padj < 0.05 as the thresholds to detect differentially expressed mRNAs. The target gene *GSEC* was identified through an exhaustive literature review. The potential target genes of lncRNA *GSEC* were predicted using the starBase website. Pearson correlation analysis was subsequently used to evaluate the correlation between lncRNA *GSEC* and IGF2BP2 expression.

### Cell culture

The normal human thyroid cell line Nthy-ori 3-1 (CL-0817) and the thyroid cancer cell lines HTh-7 (CL-0647), KTC-1 (CL-0649), and TPC-1 (CL-0643) were obtained from Procell Bio Co., Ltd. (China). The human thyroid squamous cell carcinoma cell line SW579 (SNL-451) was purchased from SUNNBIO (China). Nthy-ori 3-1 and KTC-1 cells were cultured in complete RPMI-1640 medium supplemented with 10% FBS and 1% penicillin/streptomycin (P/S). HTh-7 and TPC-1 cells were cultured in complete DMEM supplemented with 10% FBS and 1% P/S. SW579 cells were cultured in Leibovitz’s L-15 medium supplemented with 10% FBS and 1% P/S. Nthy-ori 3-1, HTh-7, KTC-1, and TPC-1 cells were cultured at 37 °C with 5% CO_2_, while SW579 cells were cultured at 37 °C in 100% air.

### Cell transfection

The pcDNA3.1 empty vector (Vector), pcDNA3.1-*GSEC* expression plasmid (LncRNA *GSEC*-OE), pcDNA3.1-*IGF2BP2* expression plasmid (*IGF2BP2-OE*), pLKO.1-Puro empty vector (sh-NC), and pLKO.1-*GLUT1* knockdown plasmid (sh-*GLUT1*) were designed and synthesized by GenePharma (China). In accordance with the manufacturer’s instructions, these plasmids were transfected into TPC-1 cells using Lipofectamine 2000 (Invitrogen, USA). After 48 hours of transfection, the culture medium was replaced with complete medium supplemented with 800 µg/mL G418 (overexpression group) or 1.5 µg/mL puromycin (knockdown group), and the medium was changed every 3 days. The screening was continued for 14 days until individual clones were visible. We selected individual clones for expansion culture and maintained the concentration at half (400 µg/mL G418 or 0.5 µg/mL puromycin). After wtable expression was verified by qRT-PCR and western blot analysis, the cells were used for subsequent experiments (passages ≤ 15 generations).

### RNA extraction and qRT-PCR

To isolate total RNA from tissue samples and cultured cells, we used Ribozol RNA extraction reagent (Thomas Scientific, USA). For the reverse transcription step, a High-Capacity cDNA Reverse Transcription Kit (Applied Biosystems, USA) was used. qRT-PCR was subsequently performed using SYBR Green PCR Master Mix (Applied Biosystems) on an Applied Biosystems real-time PCR system. β-Actin was selected as the reference gene, and the relative expression levels of the target genes were determined using the 2^-∆∆Ct^ method. The primers used are detailed in **Table 1**.

**Table 1:** Primer sequence

Gene	Sequence (5’-3’)
β*-actin*	Forward: GGCTGTATTCCCCTCCATCG
	Reverse: CCAGTTGGTAACAATGCCATGT
*GSEC*	Forward: CCCACCCTGCTCACCTAAAC
	Reverse: TTCAATTTGAGGCCCCAGGA
*GLUT1*	Forward: GGCCAAGAGTGTGCTAAAGAA
	Reverse: ACAGCGTTGATGCCAGACAG
*IGF2BP2*	Forward: AGTGGAATTGCATGGGAAAATCA
	Reverse: CAACGGCGGTTTCTGTGTC

### CCK-8 assay

Cell viability was assessed using a CCK-8 kit (Dojindo, Japan). We seeded approximately 1×10^4^ TPC-1 cells into each well of a 96-well plate. To track cell proliferation, we added 100 µL of a 10% CCK-8 solution to the cells at 0, 24, and 48 hours and incubated them for 3 hours. We then measured the absorbance at 450 nm using a Power Wave XS microplate reader (Bio-Tek, USA).

### Apoptosis analysis

Transfected TPC-1 cells were plated in 6-well plates at a density of 1×10^6^ cells per well and cultured for 48 hours at 37 °C. Apoptosis was assessed using an Annexin V-FITC/PI kit (Yeasen, China). The cells were resuspended in 100 µL of 1× binding buffer after the supernatant was removed. Afterward, 5 µL of Annexin V-FITC and 10 µL of PI were added, mixed gently, and incubated in the dark at room temperature for 10-15 minutes. Finally, 400 µL of 1× binding buffer was added, and the cells were analyzed using a BD Calibur flow cytometer.

### Western blot analysis

We extracted total protein from tissue samples and cultured cells using a RIPA Lysis Buffer Kit (Santa Cruz, USA). The proteins were separated by SDS-PAGE and transferred to PVDF membranes. The membranes were incubated with primary antibodies overnight at 4 °C. The primary antibodies used were anti-Bcl-2 (Abcam; ab182858), anti-Bax (Abcam; ab32502), anti-cleaved caspase3 (Abcam; ab32042), anti-MMP2 (Abcam; ab92536), anti-MMP9 (Abcam; ab76003), anti-GLUT1 (Abcam; ab115730), anti-hexokinase II (Abcam; ab209847), anti-PKM2 (Thermo Fisher; PA5-28700), anti-IGF2BP2 (Abcam; ab124930), and anti-β-actin (Abcam; ab8226). The membranes were then incubated with an HRP-conjugated secondary antibody (Abcam; ab205718) for 2 hours at room temperature. Protein bands were visualized using an enhanced chemiluminescence (ECL) kit (Takara, Japan), and protein expression was quantified using Quantity One software.

### EdU staining

Cell proliferation was assessed using an EdU Proliferation Kit (Abcam; ab19801). Transfected TPC-1 cells were seeded into 96-well plates at 1×10^4^ cells per well and cultured for 48 hours at 37 °C. The EdU reaction mixture provided in the kit was added to the cells, which were then incubated in the dark for 30 minutes. Excess dye was removed by washing with PBS, and cell proliferation was detected using a flow cytometer (BD Calibur, USA).

### Cell migration assay

TPC-1 cells were plated in 48-well plates and grown to confluence. Sterile 200 µL pipette tips were used to introduce scratches into the monolayer. The wells were then washed with PBS to clear away cell debris. The cells were then maintained in serum-free or low-serum medium at 37 °C. The scratch areas were imaged at 0 and 48 hours using a microscope. The scratch width and healing area were measured using ImageJ software.

### Transwell assay

Following starvation, the transfected TPC-1 cells were adjusted to an appropriate density and seeded into the upper chamber of Transwell inserts coated with Matrigel. The lower chamber contained a chemoattractant. After 48 hours of culture, the cells were fixed with 4% paraformaldehyde (PFA) for 30 minutes and stained with crystal violet for 10 minutes. The cells were subsequently washed 2-3 times with PBS. The number of cells that migrated to the lower side of the membrane was determined by counting the cells in 3-5 random fields under a microscope.

### Glucose uptake determination

TPC-1 cells subjected to various transfection strategies were incubated with 100 µM 2NBDG (APExBIO, USA) at 37 °C for 30 minutes to assess glucose uptake. After incubation, the cells were centrifuged at 1000 rpm for 5 minutes, and excess 2NBDG was removed by washing with PBS. Glucose uptake was then analyzed using a flow cytometer (BD Calibur, USA).

### Lactate concentration assessment

To determine the lactate levels in the supernatant of the transfected TPC-1 cells, we used a Lactate Assay Kit (Beyotime, China). We collected the supernatant and added 50 µL to each well of a 96-well plate. We then added 50 µL of WST-8 colorimetric working solution to each well, mixed well, and incubated the plate at 37 °C in the dark for 30 minutes. After incubation, we measured the absorbance at 450 nm and calculated the lactate concentration based on a standard curve.

### *In vivo* assay

We obtained 12 male BALB/c nude mice (5-6 weeks old) from GemPharmatech (China) and acclimated them for two weeks under standard conditions (12-hour light/dark cycle; 55% relative humidity). Human PTC cells, transfected with either a control vector or lncRNA *GSEC*-OE, were injected subcutaneously into the flanks of the mice at a dose of 2 × 10^7^ cells per mouse to create a PTC xenograft model. The tumor volumes were measured every five days, and the mice were sacrificed after 20 days. The tumors were then excised, weighed, photographed, and fixed in 4% paraformaldehyde for histological analysis. For the metastasis model, luciferase-labeled PTC cells were injected into the tail veins of mice, and their *in vivo* growth and metastasis were tracked using a small-animal *in vivo* imaging system at designated time points.

### Histological analysis

Patient samples or tumor tissues collected from tumor-bearing mice were fixed in 4% PFA for 24 hours, embedded in paraffin and cut into 5 µm sections. Hematoxylin and eosin (H&E) staining and TUNEL staining were conducted to explore pathological changes and proliferation in the tumors. IHC staining was performed to detect the expression of anti-cleaved caspase3 (Abcam; ab32042) and anti-IGF2BP2 (Abcam; ab124930) in the tumor tissue.

### Statistical analysis

All experiments were conducted in triplicate to ensure reliability. The results are presented as the mean ± standard deviation (SD). Statistical analyses were performed using SPSS v.19.0 software (IBM, USA). Student’s *t* test was used to compare two groups, while one-way ANOVA was used for comparisons among multiple groups. Pearson correlation analysis was performed to evaluate the correlation between *GSEC* and IGF2BP2 expression levels. Significance was defined as *P* < 0.05 (*) or *P* < 0.01 (**).

## RESULTS

### Reduced expression of lncRNA *GSEC* in thyroid cancer

**Figure 1:**
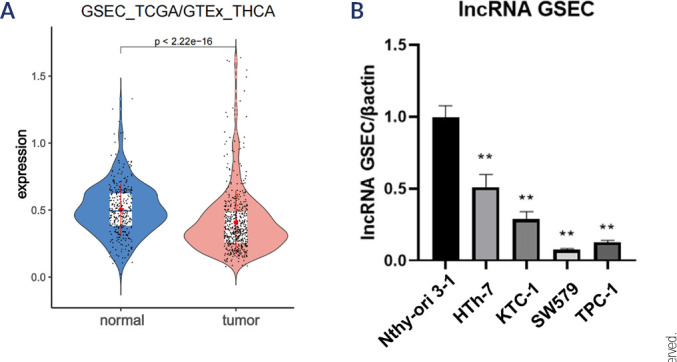
Reduced expression of LncRNA *GSEC* in thyroid cancer. (**A**) Expression levels of LncRNA *GSEC* in thyroid cancer obtained from the TCGA database; (**B**) qRT-PCR was performed to detect LncRNA *GSEC* expression in human normal thyroid cells (Nthy-ori 3-1) and human thyroid cancer cell lines (HTh-7, KTC-1, SW579, and TPC-1). *P < 0.05; **P < 0.01.

We initiated our research by leveraging bioinformatics tools to examine lncRNA *GSEC* expression in thyroid cancer. Compared with that in normal thyroid tissues, *GSEC* expression in thyroid cancer tissues was reduced (*P*<2.22e-16) (**Figure 1A**). Subsequent analysis of *GSEC* expression in normal human thyroid cells (Nthy-ori 3-1) and diverse thyroid cancer cell lines (HTh-7, KTC-1, TPC-1, and SW579) revealed a marked disparity. The percentage of *GSEC* was notably greater in Nthy-ori 3-1 cells but markedly lower in the cancer cell lines (**Figure 1B**). These results indicate that *GSEC* expression is generally downregulated in thyroid cancer cells. In this study, we focused on further investigating the potential role of lncRNA *GSEC* in TPC-1 cells.

### Overexpression of lncRNA *GSEC* inhibits PTC progression

**Figure 2:**
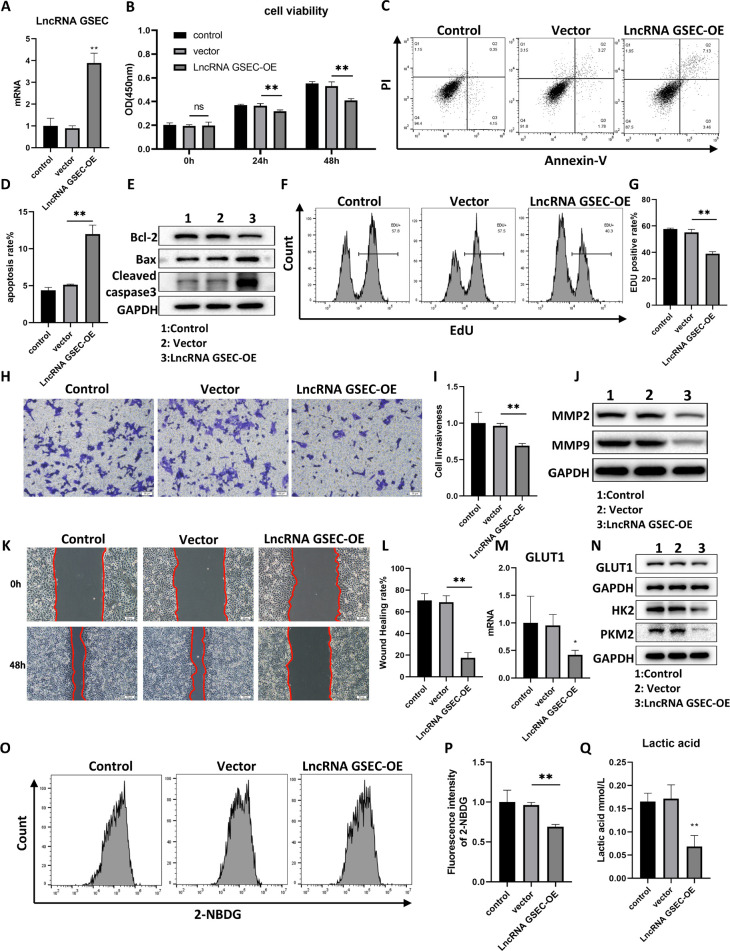
Overexpression of LncRNA *GSEC* inhibits PTC progression. (**A**) qRT-PCR analysis showed increased LncRNA *GSEC* mRNA levels in TPC-1 cells transfected with LncRNA *GSEC*-OE; (**B**) CCK-8 assay revealed decreased cell viability in TPC-1 cells at different time points; (**C-D**) Annexin-V/PI staining showed increased apoptosis levels in TPC-1 cells transfected with LncRNA *GSEC*-OE; (**E**) WB analysis showed changes in Bcl-2, Bax, and Cleaved caspase-3 protein levels in TPC-1 cells; (**F-G**) EdU staining revealed reduced proliferation in TPC-1 cells; (**H-I**) Transwell assay showed decreased invasion ability in TPC-1 cells; (**J**) WB analysis showed changes in MMP2 and MMP9 protein levels in TPC-1 cells; (**K-L**) Wound healing assay showed reduced migration ability in TPC-1 cells; (**M**) qRT-PCR analysis showed changes in *GLUT1* expression in TPC-1 cells transfected with LncRNA *GSEC*-OE; (**N**) WB analysis showed changes in GLUT1, HK, and PKM2 protein levels in TPC-1 cells; (**O-P**) Flow cytometry showed changes in glucose uptake ability in TPC-1 cells.; (**Q**) Lactate levels in the supernatant of TPC-1 cells were measured. *P < 0.05; **P < 0.01.

To elucidate the impact of *GSEC* on PTC progression, we overexpressed the *GSEC* gene in TPC-1 cells and successfully established a cell line with high *GSEC* expression, designated lncRNA *GSEC*-OE (**Figure 2A**). We first examined the effect of *GSEC* expression on the viability of TPC-1 cells. The results of the CCK-8 assay (**Figure 2B**) revealed that after the culture period exceeded 24 hours, the viability of TPC-1 cells overexpressing *GSEC* was significantly lower than that of the control and empty vector (Vector) groups. Additionally, apoptosis assays (**Figures 2C-D**) revealed that the apoptosis rate of TPC-1 cells increased significantly upon overexpression of *GSEC*, exceeding 10%. After *GSEC* was overexpressed, western blot analysis revealed a significant decrease in the expression of the antiapoptotic protein Bcl-2, along with a marked increase in the expression of the proapoptotic proteins Bax and cleaved caspase3 (**Figure 2E**). This shift in protein expression suggests that *GSEC* overexpression can trigger apoptosis. To further assess cell proliferation, we used EdU staining and flow cytometry. The results revealed that the percentage of EdU-positive cells was significantly lower in the lncRNA *GSEC*-OE group than in the control and vector groups (**Figures 2F-G**), indicating that *GSEC* overexpression inhibited cell proliferation. We also investigated the invasive and migratory capabilities of the cells. Using Transwell assays and western blot analysis, we observed that the invasive potential of cells with elevated *GSEC* expression substantially decreased (**Figures 2H-I**). This reduction was accompanied by a notable decrease in the expression of matrix metalloproteinase 2 (MMP2) and matrix metalloproteinase 9 (MMP9) (**Figure 2J**). Furthermore, wound-healing assays revealed that the migration rate of the lncRNA *GSEC*-OE group was only approximately 20% at 48 hours, whereas that of the control and vector groups was nearly 70% (**Figures 2K-L**). These results highlight the inhibitory effect of *GSEC* overexpression on cell invasion and migration.

Given that metabolic reprogramming is a hallmark of cancer progression, we explored how *GSEC* expression affects glycolysis. Our qRT-PCR analysis revealed that overexpressing *GSEC* significantly reduced *GLUT1* mRNA levels (**Figure 2M**). Western blot analysis further revealed that the protein levels of GLUT1, HK2, and PKM2 were notably lower in *GSEC*-overexpressing cells than in control and vector-transfected cells (**Figure 2N**). These proteins are pivotal for glycolysis, and their reduced expression suggests that *GSEC* overexpression may disrupt the glycolytic pathway in cancer cells. The overexpression of *GSEC* notably affected cancer cell metabolism. Specifically, flow cytometry using 2-NBDG revealed a significant reduction in glucose uptake by cancer cells (**Figures 2O-P)**. This metabolic shift was further evidenced by a marked decrease in lactate production in the cell supernatant (**Figure 2Q**). Collectively, these results indicate that *GSEC* likely influences cancer progression by affecting glycolysis.

### Overexpression of lncRNA *GSEC* downregulates IGF2BP2 expression

**Figure 3:**
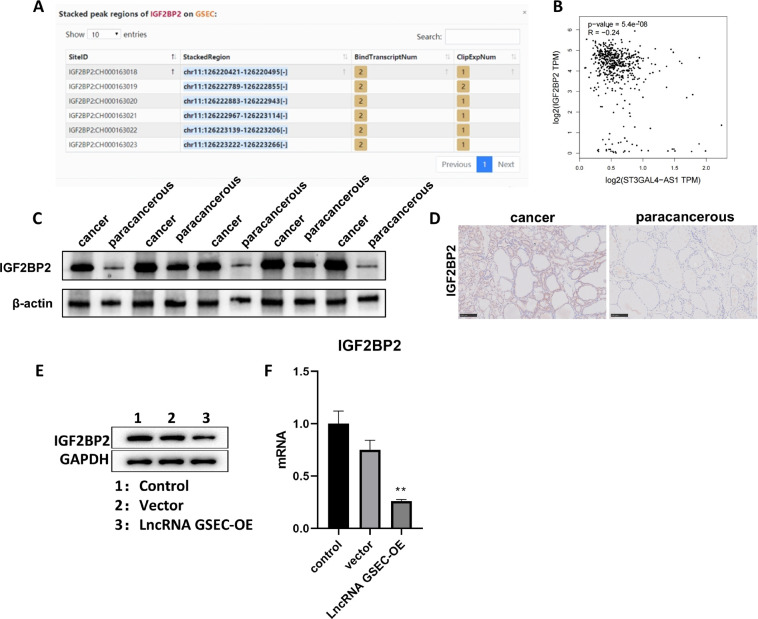
Overexpression of LncRNA *GSEC* downregulates IGF2BP2 expression. (**A**) Predicted target genes of LncRNA *GSEC* identified via Starbase; (**B**) Correlation analysis between LncRNA *GSEC* and *IGF2BP2* expression; (**C-D**) WB and IHC staining examined IGF2BP2 expression in tumor tissues and paired adjacent non-tumor tissues; (E-F) qRT-PCR and WB analysis revealed decreased *IGF2BP2* mRNA and protein levels in TPC-1 cells overexpressing *GSEC*. **P < 0.01.

To elucidate the molecular underpinnings of how lncRNA *GSEC* affects cancer progression, we used the starBase platform to pinpoint IGF2BP2 as a likely target regulated by *GSEC* (**Figure 3A**). Further scrutiny via the GEPIA database revealed a pronounced negative correlation between *GSEC* and *IGF2BP2* expression in PTC (P value = 5.4e-08; R = -0.24) (**Figure 3B**). Western blot analysis of 5 paired PTC samples and their adjacent nontumor tissues revealed significantly elevated expression of IGF2BP2 in tumor tissues (**Figure 3C**). We subsequently performed IHC staining on 5 pairs of samples and detected abnormal upregulation of IGF2BP2 in cancer tissues (**Figure 3D**). To corroborate this finding at the cellular level, we overexpressed *GSEC* and observed a substantial decrease in both the mRNA and protein levels of IGF2BP2 (**Figures 3E-F**). Collectively, these data suggest that the effects of lncRNA *GSEC* may involve targeting and suppressing IGF2BP2 expression.

### IGF2BP2 drives cancer progression via GLUT1 upregulation

**Figure 4:**
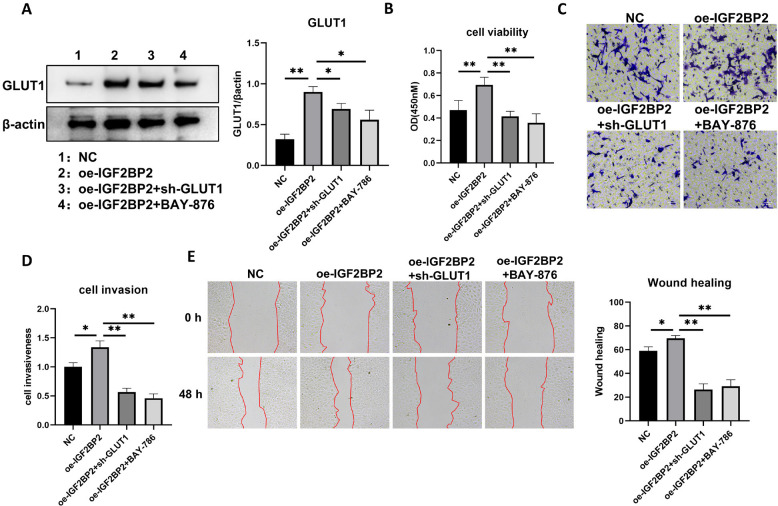
IGF2BP2 promotes cancer progression by upregulating GLUT1 expression. (**A**) WB analysis of GLUT1 protein expression in treated cells; (**B**) CCK-8 assay measuring cell viability of TPC-1 cells under different treatments; (**C, D**) Transwell assay evaluating cell invasive capability; (**E**) Wound-healing assay assessing cell migratory ability. *P < 0.05; **P < 0.01.

IGF2BP2 can increase GLUT1 expression by stabilizing its mRNA (^16^). Based on this mechanism, we hypothesized that lncRNA *GSEC* might influence glycolytic levels through an IGF2BP2-mediated GLUT1 regulatory mechanism. To investigate whether IGF2BP2 regulates *GLUT1* expression in PTC, we established *IGF2BP2* overexpression and knockdown models in TPC-1 cells combined with intervention with the GLUT1 inhibitor BAY-876. Western blot results (**Figure 4A**) revealed that *IGF2BP2* overexpression significantly upregulated GLUT1 protein levels, whereas *GLUT1* knockdown in the context of IGF2BP2 overexpression markedly reduced its expression, suggesting that IGF2BP2 positively regulates GLUT1. Further functional assays demonstrated that *IGF2BP2* overexpression significantly increased cell viability, whereas *GLUT1* knockdown or BAY-876 treatment reversed this effect (**Figure 4B**), indicating that IGF2BP2 critically influences cancer cell activity via GLUT1 regulation. In the migration and invasion assays, the Transwell results (**Figures 4C and D**) revealed that compared with control cells, oe-IGF2BP2 cells exhibited significantly increased invasive capacity. However, GLUT1 knockdown or BAY-876 treatment substantially suppressed this pro-invasive effect in oe-IGF2BP2 cells. A similar trend was observed in the wound-healing assay (**Figure 4E**), where IGF2BP2 promoted cell migration, whereas GLUT1 knockdown or inhibition significantly attenuated this effect. In summary, these findings demonstrate that IGF2BP2 enhances the proliferation, migration, and invasion of TPC-1 cells by upregulating GLUT1 expression, suggesting that the IGF2BP2/GLUT1 axis plays a critical role in PTC progression.

### LncRNA *GSEC* regulates cancer cell glucose uptake via IGF2BP2

**Figure 5:**
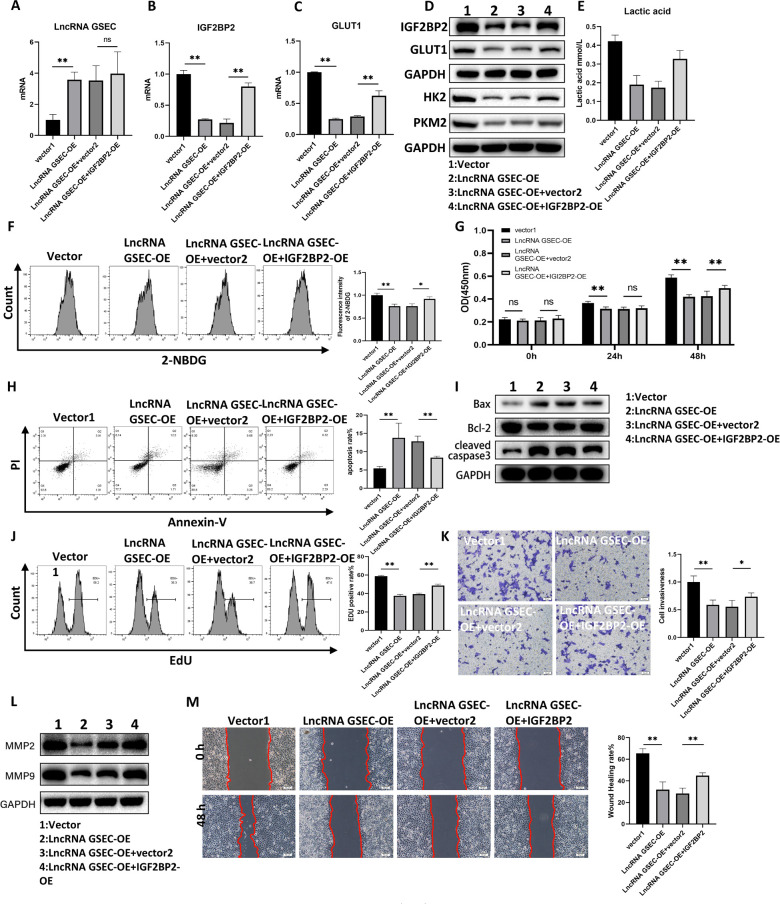
LncRNA *GSEC* regulates cancer cell glucose uptake via IGF2BP2. (**A-C**) qRT-PCR analysis of mRNA levels of *GSEC, IGF2BP2*, and *GLUT1* in TPC-1 cells; (**D**) WB analysis of protein expression levels of IGF2BP2, GLUT1, HK, and PKM2 in TPC-1 cells; (**E**) Lactate levels in the supernatant of TPC-1 cells; (**F**) Glucose uptake in TPC-1 cells assessed by flow cytometry; (**G**) Cell viability of TPC-1 cells at different time points assessed by CCK-8 assay; (**H**) Apoptosis levels in TPC-1 cells assessed by Annexin-V/PI staining; (**I**) WB analysis of Bcl-2, Bax, and Cleaved caspase-3 protein levels in TPC-1 cells; (**J**) Proliferation of TPC-1 cells assessed by EdU staining; (**K**) Invasion ability of TPC-1 cells assessed by Transwell assay; (**L**) WB analysis of MMP2 and MMP9 protein levels in TPC-1 cells; (**M**) Migration ability of TPC-1 cells assessed by wound healing assay. *P < 0.05; **P < 0.01.

Having established that lncRNA *GSEC* upregulates IGF2BP2 expression and that IGF2BP2 promotes cancer progression through GLUT1, we further investigated whether *GSEC* exerts its regulatory effects on glycolytic metabolism and malignant phenotypes in an *IGF2BP2*-dependent manner. We established four cellular models-vector1, lncRNA *GSEC*-OE, lncRNA *GSEC*-OE+vector2, and lncRNA *GSEC*-OE+IGFB2BP2-OE-and measured the expression levels of relevant genes using qRT-PCR (**Figures 5A-C**). We found that *GSEC* overexpression significantly reduced the expression of IGFB2BP2 and GLUT1. However, when we transfected these cells with *IGFB2BP2*-OE, the expression of both *IGFB2BP2* and *GLUT1* was significantly restored (**Figures 5B-C**). This trend was also confirmed at the protein level. Western blot analysis revealed that the protein levels of HK2 and PKM2, as well as those of IGF2BP2 and GLUT1, were restored after *IGF2BP2*-OE reconstitution (**Figure 5D**). Systemic lactate production and glucose uptake were enhanced following *IGF2BP2*-OE transfection (**Figures 5E-F**). These results suggest that lncRNA *GSEC* inhibits glycolysis by downregulating the expression of IGF2BP2.

We evaluated the malignancy of cancer cells by examining their proliferation, invasion, and metastasis capabilities. The results of the CCK-8 assay revealed that compared with the lncRNA *GSEC*-OE + *IGFB2BP2*-OE group, the lncRNA *GSEC*-OE group had significantly lower cell viability. These findings suggest that *GSEC* expression inhibits cell proliferation, whereas IGFB2BP2 expression can counteract this effect (**Figure 5G**). The results of apoptosis assays further revealed that IGFB2BP2 expression weakened *GSEC*-induced apoptosis (**Figures 5H-I**). These findings highlight the potential therapeutic implications of targeting *GSEC* and IGFB2BP2 in cancer treatment. EdU staining also revealed a similar trend, with the proliferation rate of cells in the lncRNA *GSEC*-OE group being significantly lower than that in the lncRNA *GSEC*-OE + *IGFB2BP2*-OE group (**Figure 5J**). Consistent with these findings, invasion assays (**Figures 5K-L**) and wound-healing experiments (**Figure 5M**) revealed that the invasive and migratory abilities of the lncRNA *GSEC*-OE + *IGFB2BP2*-OE cells were significantly enhanced. These results collectively suggest that lncRNA *GSEC* can inhibit cancer progression by downregulating the expression of IGFB2BP2, thereby affecting glycolysis in cancer cells.

### Overexpression of lncRNA *GSEC* suppresses PTC progression in nude mice

**Figure 6:**
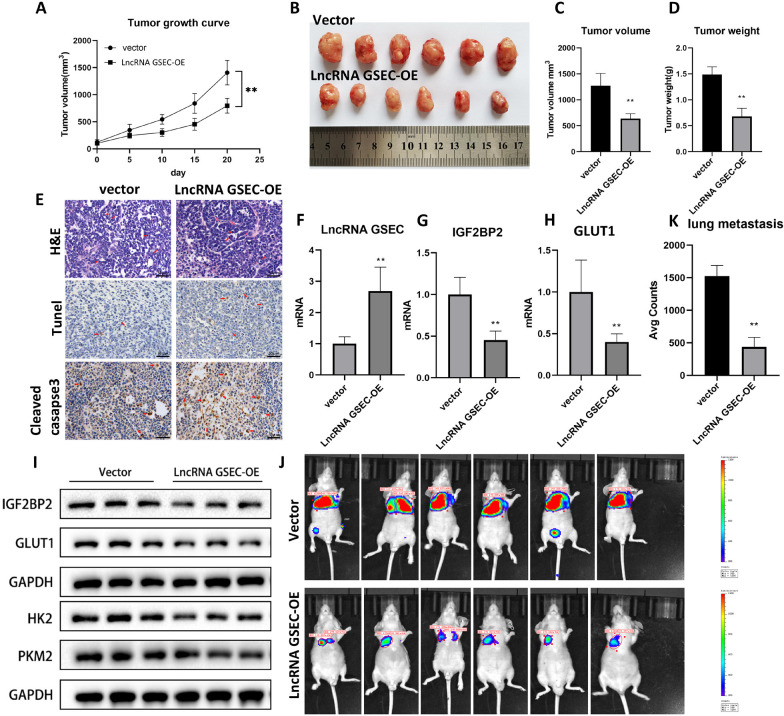
Overexpression of LncRNA *GSEC* suppresses TPC progression in nude mice. (**A**) Changes in tumor volume in mice during treatment; (**B**) Representative images of tumors excised from mice; (**C-D**) Tumor volume (**C**) and weight (**D**) at the end of treatment; (**E**) Histological staining of tumor tissues with H&E, TUNEL, and Cleaved caspase-3; (**F-H**) qRT-PCR analysis revealed mRNA expression levels of LncRNA *GSEC, IGF2BP2*, and *GLUT1* in tumor tissues; (**I**) WB analysis showed protein levels of IGF2BP2, GLUT1, HK2, and PKM2 in tumor tissues; (**J-K**) Representative bioluminescent images (**J**) and quantitative analysis of lung metastasis (**K**) in mice. *P < 0.05; **P < 0.01.

To assess the *in vivo* effects of *GSEC* on cancer progression, we established a mouse PTC xenograft model. Nude mice were randomly assigned to one of two groups, and TPC-1 cells transfected with either vector or lncRNA *GSEC*-OE were injected subcutaneously into their right flanks. Tumor growth was closely monitored throughout the experiment. As shown in **Figures 6A-D**, tumors in the control group grew rapidly and were significantly larger, whereas those in the lncRNA *GSEC*-OE group grew more slowly and were smaller at the end of the experiment. H&E staining of tumor tissues revealed that while the tumor architecture was intact in the vector group, the tumor architecture was noticeably necrotic in the lncRNA *GSEC*-OE group (**Figure 6E**). These findings were corroborated by TUNEL staining, which revealed a higher incidence of apoptosis in the lncRNA *GSEC*-OE group (**Figure 6E**), suggesting that *GSEC* overexpression effectively inhibits PTC growth. Furthermore, *GSEC* mRNA expression was significantly upregulated in the lncRNA *GSEC*-OE group, verifying the successful overexpression of *GSEC* (**Figure 6F**). Moreover, the downregulation of IGF2BP2, GLUT1, HK2, and PKM2 expression in the lncRNA *GSEC*-OE group indicated that *GSEC* overexpression suppressed glycolysis in cancer cells (**Figures 6G-I**). To assess the impact of *GSEC* expression on cancer metastasis, we established an *in vivo* PTC metastasis model by intravenously injecting luciferase-labeled TPC-1 cells into mice. Live imaging revealed that the vector group exhibited robust lung metastasis, whereas the lncRNA *GSEC* group showed significantly reduced metastatic activity, as evidenced by lower avg counts (**Figures 6J and K**). These results indicate that *GSEC* overexpression can effectively diminish the metastatic potential of tumor cells *in vivo*.

## DISCUSSION

In our study, we initially noted a pronounced downregulation of lncRNA *GSEC* in PTC. We found that *GSEC* expression can curb cancer cell progression and glycolysis. Using starBase, we identified IGF2BP2 as a downstream target of *GSEC*. Our data show that *GSEC* could impede glycolytic reprogramming and malignant progression in cancer cells by inhibiting the IGF2BP2/GLUT1 axis. Our findings underscore the pivotal role of lncRNA *GSEC* in PTC progression and shed new light on the metabolic regulatory mechanisms in PTC.

LncRNA *GSEC*, also known as *DCPS-AS1* or *ST3GAL4-AS1*, is a long noncoding RNA containing a G-quadruplex structure that was first identified by Matsumura and cols. (^13^) in colon cancer. Previous studies have shown that *GSEC* is highly expressed in various types of tumors, such as colon cancer and lung adenocarcinoma, and can participate in tumor occurrence and progression through multiple molecular mechanisms (^12^,^13^). Among them, *GSEC* can regulate the availability of miRNAs through a competitive endogenous RNA (ceRNA) mechanism, thereby affecting the expression and function of downstream target genes (^14^). For example, in triple-negative breast cancer (TNBC), *GSEC* upregulates the expression of AXL by adsorbing miR-202-5p, thereby promoting the proliferation, migration, and invasion of tumor cells (^17^). In addition, increasing evidence suggests that *GSEC* is involved in regulating cellular metabolic reprogramming. For example, in sepsis models, *GSEC*s can promote inflammatory activation in neutrophils by regulating PFKFB3-mediated glycolytic metabolism (^15^). The results of this study suggest that *GSEC*-regulated glycolysis may be an important mechanism involved in disease progression. Notably, the above studies generally support the role of *GSEC* in promoting cancer or pathological progression in most diseases or tumors. However, an increasing number of studies have shown that the biological functions of lncRNAs often exhibit significant tissue specificity and tumor microenvironment dependence and that the same lncRNA may play vastly different regulatory roles in different tumor types (^18^). For example, the sialyltransferase ST3GAL4 can promote tumor progression by enhancing aerobic glycolysis (^19^). As the antisense transcript of *ST3GAL4*, lncRNA *GSEC* may interfere with the translation process of ST3GAL4 by forming a double-stranded RNA structure with its sense transcript (^20^-^22^), thereby inhibiting the activation effects of sialylation-related glucose metabolism. In this study, we observed a significantly low expression level of *GSEC* in PTC, and its overexpression significantly inhibited the proliferation, invasion, and migration abilities of cancer cells. Further mechanistic studies have shown that *GSEC* can weaken the glucose metabolism process in cancer cells by inhibiting the expression of IGF2BP2 and downregulating GLUT1 levels. These results indicate that the role of *GSEC* in PTC differs from its reported procancer function in other tumors, but that it exerts an anticancer effect by inhibiting the IGF2BP2/GLUT1 axis. This study not only reveals the unique biological function of *GSEC* in PTC but also provides a new perspective for understanding its functional differences in different tumor types and provides a potential molecular basis for metabolic targeted therapy for PTC.

IGF2BP2, an RNA-binding protein, is involved in multiple cellular processes (^23^). It binds to and stabilizes various mRNAs, thereby influencing gene expression at the posttranscriptional level and affecting essential cellular functions such as proliferation, differentiation, and metabolism (^24^). While IGF2BP2 is crucial for maintaining cellular homeostasis under normal conditions, its overexpression in cancer can drive tumor progression and malignancy. IGF2BP2 is often upregulated in various cancers and functions as an oncogene (^25^,^26^). In head and neck squamous cell carcinoma, it promotes tumor cell proliferation and growth through the miR-98-5p/PI3K/Akt signaling pathway. IGF2BP2 also appears to be a key regulator of glycolysis in cancer cells (^27^). In pancreatic ductal adenocarcinoma, IGF2BP2 plays a significant role in enhancing cancer cell metabolism, as reported by Huang and cols. (^16^). Specifically, IGF2BP2 binds to *GLUT1* mRNA and stabilizes it, leading to increased GLUT1 expression; this stabilization increases the degree of aerobic glycolysis in cells, which supplies them with the energy and metabolic precursors required for rapid proliferation; additionally, this mechanism alters the cellular metabolic microenvironment, driving tumor progression. A recent study by Wang and cols. (^28^) has shed light on the role of IGF2BP2 in PTC. Their analysis of PTC transcriptome data from the TCGA revealed that IGF2BP2 is significantly overexpressed in PTC tissues and that its overexpression is strongly correlated with disease-free survival and clinical outcomes in PTC patients. These findings from the two studies suggest that IGF2BP2 may increase glycolysis in cancer cells, potentially fuelling PTC progression. Our investigation further illuminated this area. In terms of the role of lncRNA *GSEC* in PTC, we observed that *GSEC* expression could decrease IGF2BP2 levels. Its downregulation, in turn, destabilized GLUT1, ultimately curbing the glucose uptake of PTC cells. Our findings not only elucidate how IGF2BP2 is regulated in PTC but also paves the way for new cancer therapies targeting IGF2BP2.

In summary, our research revealed the critical role of lncRNA *GSEC* in the progression of PTC. Our findings show that the expression of *GSEC* is markedly downregulated in PTC. The overexpression of *GSEC* inhibits the IGF2BP2/GLUT1 axis, effectively blocking glycolytic reprogramming and the subsequent malignancy of cancer cells. This discovery offers a fresh perspective on metabolic regulation in PTC and points to a potential molecular target for treatment. However, this study has several limitations. First, although we have shown through functional experiments and molecular-level evidence that lncRNA *GSEC* can reduce glucose uptake and lactate production by inhibiting the IGF2BP2/GLUT1 axis, thereby inhibiting cancer cell glucose metabolism reprogramming, there is currently a lack of direct metabolic flow evidence for its regulation of anaerobic glycolysis, which still needs to be further validated through metabolomics or isotope tracing experiments. In addition, the specific molecular mechanism through which lncRNA *GSEC* inhibits IGF2BP2 expression, whether it plays a role through RNA-protein interactions, posttranscriptional regulation, or other indirect channels, remains to be further studied. Finally, as multifunctional regulatory molecules, lncRNAs often have a wide spectrum of target genes. Although this study focused on the key role of the IGF2BP2/GLUT1 axis in PTC glucose metabolism reprogramming, we did not systematically evaluate the regulatory effects of *GSEC* on other metabolic pathways, nor did we explore its functional differences across different PTC cell subtypes or tumor microenvironments. These are the research directions that we need to improve upon in the future.

## Data Availability

datasets related to this article will be avail-able upon request to the corresponding author.
